# Supplementary material for: Camera-trapping estimates of the relative population density of Sympetrum dragonflies: application to multihabitat users in agricultural landscapes
Source: PeerJ. 2023 Feb 28;11:e14881. doi: 10.7717/peerj.14881 (PMC9983425; doi:10.7717/peerj.14881)
Supplement: Figure S1 — Each study plot consists of the rice-paddy fields enclosed by solid red line(s). However, the camera trap locations and the study plot of Kanaya in 2018 was shown by green points and dashed red line, respectively. The background aerial photo maps except for Sugaya were obtained from (Geospatial Information Authority of Japan, 2016) following the Geospatial Information Authority of Japan Website Terms of Use (https://www.gsi.go.jp/ENGLISH/page_e30286.html, accessed 2022-11-11), which are compatible with the Creative Commons Attribution License 4.0 (https://creativecommons.org/licenses/by/4.0/legalcode, accessed 2022-11-11). The background aerial photo of Sugaya, at which no aerial photo after 2000s was available, was captured by an author (Hirofumi OUCHI) using the Mavic2 pro (DJI Co., Ltd.) in 2022 and georeferenced using QGIS version 3.22.9. Please note that the date of photograph was not overlapped with the camera trap survey term and did not show the phenological status of the plots during our study. Map data: Geospatial Information authority of Japan, 2016. GSI tile: the latest aerial photograph throughout Japan (seamless). https://maps.gsi.go.jp/development/ichiran.html, accessed 2022-08-05 (in Japanese). [file peerj-11-14881-s002.pdf]

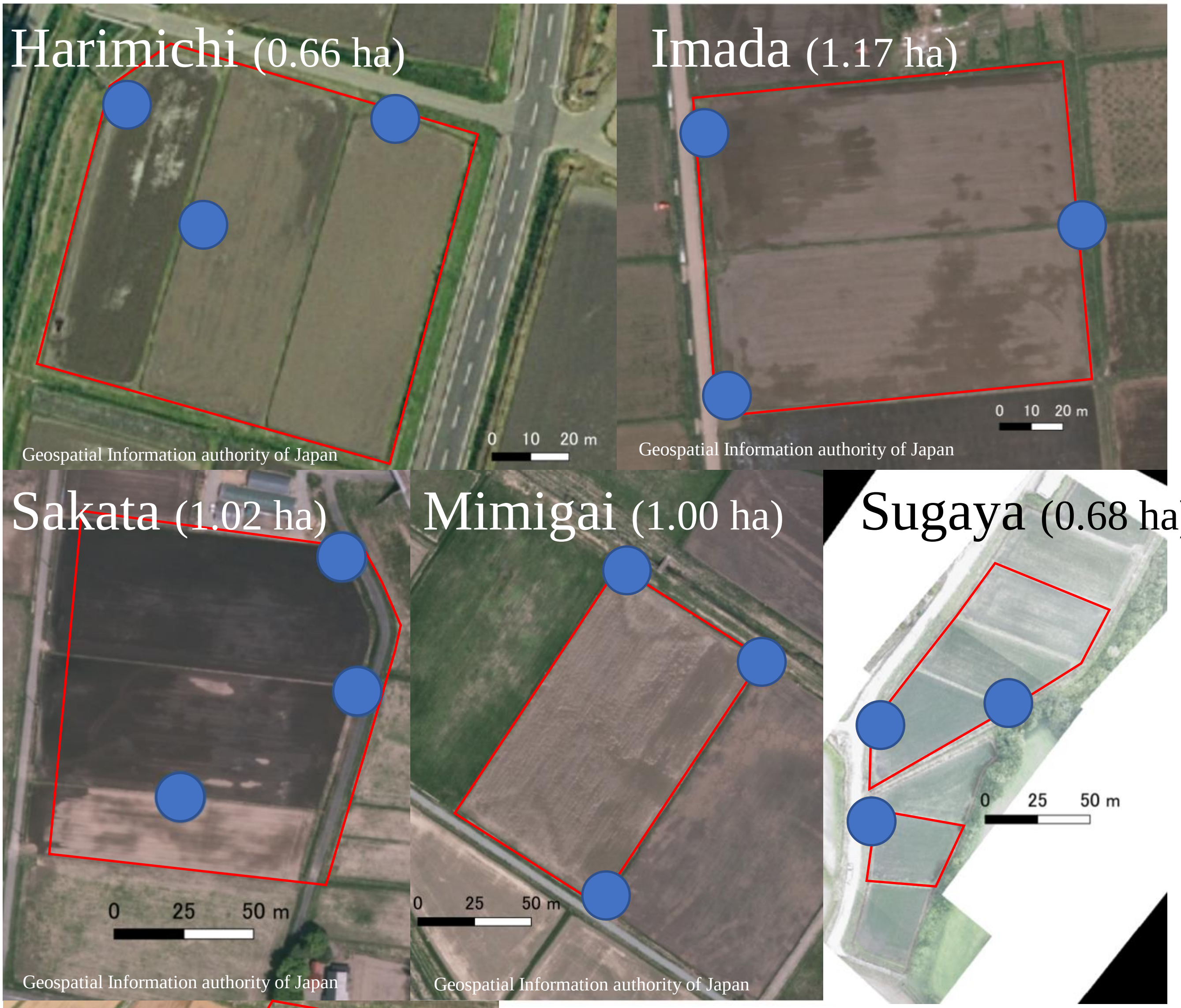

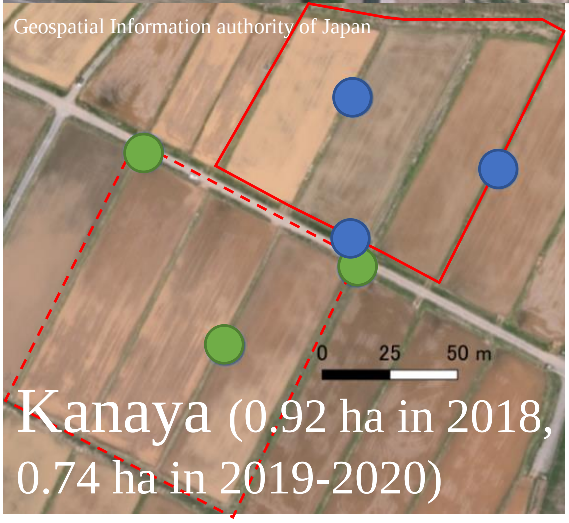

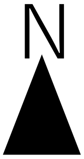

Fig. S1 Map of the camera trap locations (shown by blue points). Each study plot consists of the rice-paddy fields enclosed by solid red line(s). However, the camera trap locations and the study plot of Kanaya in 2018 was shown by green points and dashed red line, respectively. The background aerial photo maps except for Sugaya were obtained from Geospatial Information Authority of Japan (2016) following the Geospatial Information Authority of Japan Website Terms of Use (https://www.gsi.go.jp/ENGLISH/page_e30286.html, accessed 2022-11-11), which are compatible with the Creative Commons Attribution License 4.0 (https://creativecommons.org/licenses/by/4.0/legalcode, accessed 2022-11-11). The background aerial photo of Sugaya, at which no aerial photo after 2000s was available, was captured by an author (Hirofumi OUCHI) using the Mavic2 pro (DJI Co., Ltd.) in 2022 and georeferenced using QGIS version 3.22.9. Please note that the date of photograph was not overlapped with the camera trap survey term and did not show the phenological status of the plots during our study.

Map data: Geospatial Information authority of Japan, 2016. GSI tile: the latest aerial photograph throughout Japan (seamless). https://maps.gsi.go.jp/development/ichiran.html, accessed 2022-08-05 (in Japanese).
