# Supplementary material for: Camera-trapping estimates of the relative population density of Sympetrum dragonflies: application to multihabitat users in agricultural landscapes
Source: PeerJ. 2023 Feb 28;11:e14881. doi: 10.7717/peerj.14881 (PMC9983425; doi:10.7717/peerj.14881)
Supplement: Figure S2 — (A) Version 1 is characterized by the processor part enclosed in a grey PVC pipe. (B) Version 2 is characterized by a transparent PVC pipe surrounding the processor part and the extended switch. (C) Version 3 is characterized by a translucent polypropylene bottle for salad dressing enclosing the processor part. All the photographs were taken by an author (Akira Yoshioka). Yoshioka, A., Shimizu, A., Oguma, H., Kumada, N., Fukasawa, K., Jingu, S., Kadoya, T., 2020. Development of a camera trap for perching dragonflies: A new tool for freshwater environmental assessment. PeerJ 8, e9681. https://doi.org/10.7717/peerj.9681 [file peerj-11-14881-s003.pdf]

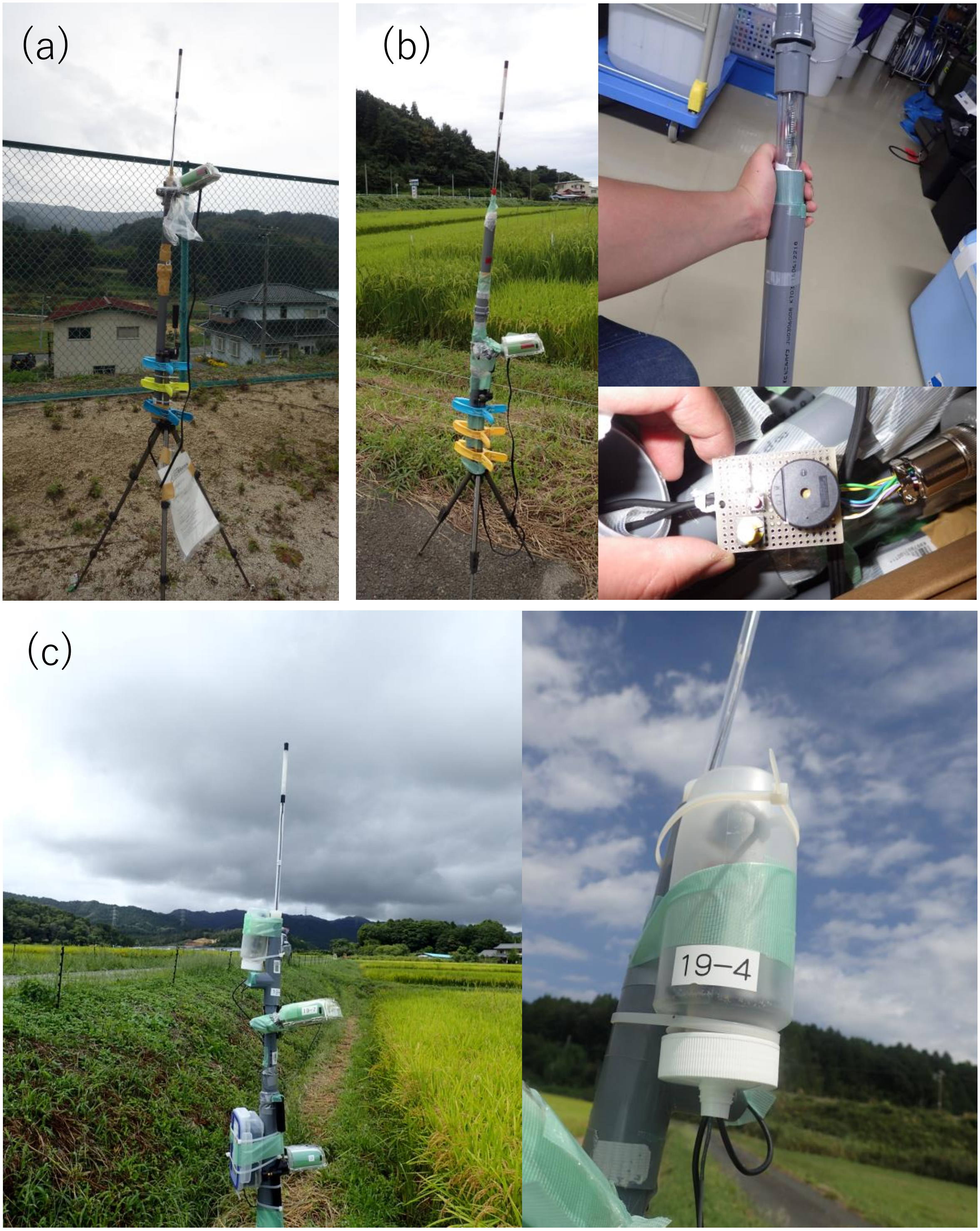

Fig. S2 Three version of the original camera traps (Yoshioka et al. 2020) applied to the camera trapping survey in the rice paddy landscapes. (a) The version 1 is characterized by the processor part enclosed in a grey PVC pipe. (b) The version 2 is characterized by a transparent PVC pipe surrounding the processor part and the extended switch. (c) The version 3 is characterized by a translucent polypropylene bottle for salad dressing enclosing the processor part. All the photographs were taken by an author (Akira YOSHIOKA).

Yoshioka, A., Shimizu, A., Oguma, H., Kumada, N., Fukasawa, K., Jingu, S., Kadoya, T., 2020. Development of a camera trap for perching dragonflies: A new tool for freshwater environmental assessment. PeerJ 8, e9681. https://doi.org/10.7717/peerj.9681
